# Autistic adults exhibit highly precise representations of others’ emotions but a reduced influence of emotion representations on emotion recognition accuracy

**DOI:** 10.1038/s41598-023-39070-0

**Published:** 2023-07-22

**Authors:** Connor T. Keating, Eri Ichijo, Jennifer L. Cook

**Affiliations:** 1 School of Psychology, University of Birmingham, Birmingham, UK; 2 Department of Experimental Psychology, University of Oxford, Oxford, UK

**Keywords:** Psychology and behaviour, Human behaviour

## Abstract

To date, studies have not yet established the mechanisms underpinning differences in autistic and non-autistic emotion recognition. The current study first investigated whether autistic and non-autistic adults differed in terms of the precision and/or differentiation of their visual emotion representations and their general matching abilities, and second, explored whether differences therein were related to challenges in accurately recognizing emotional expressions. To fulfil these aims, 45 autistic and 45 non-autistic individuals completed three tasks employing dynamic point light displays of emotional facial expressions. We identified that autistic individuals had more precise visual emotion representations than their non-autistic counterparts, however, this did not confer any benefit for their emotion recognition. Whilst for non-autistic people, non-verbal reasoning and the interaction between precision of emotion representations and matching ability predicted emotion recognition, no variables contributed to autistic emotion recognition. These findings raise the possibility that autistic individuals are less guided by their emotion representations, thus lending support to Bayesian accounts of autism.

## Introduction

Autism spectrum disorder (ASD) is a neurodevelopmental condition, characterized by restricted and repetitive interests and difficulties with social communication and interaction^1^. While not considered a core diagnostic feature, emotion recognition has been a topic of interest in autism research for over 30 years because it is often thought that challenges in this area might be an underlying cause for social difficulties. However, findings in this literature are famously mixed (see Ref.^2^ for a review): some studies find differences in emotion recognition between autistic (identity-first terminology is used throughout this manuscript in line with the preferences of the majority of the autistic community^3,4^) and non-autistic people, some studies find no differences and some find quite specific difficulties (e.g. with angry expressions^5–11^). In this literature it is often the case that “emotion recognition” is treated as a unitary or modular ability. However, recent work has begun to elucidate several component processes that contribute to individual differences in emotion recognition. Here we 1) compare autistic and non-autistic individuals on various abilities which we know to be involved in (non-autistic) emotion recognition, and 2) test whether these processes also contribute to emotion recognition in autistic adults. Understanding the extent to which different tasks rely on these factors might help us to understand the variability in findings in this literature.

Recent work has highlighted that a person’s internal templates—that is the way one pictures emotional expressions in the “minds’ eye” (also known as a *visual representations* of emotion; see Ref.^12–16^)—are important contributors to emotion recognition accuracy^17^. Signal detection theory (see Ref.^18^) tells us that at least two properties of visual representations should predict emotion recognition accuracy: precision and differentiation. That is, a ‘signal’ distribution and a ‘noise’ distribution that are both imprecise (wide) and indistinct (overlapping) provide low sensitivity to discriminate between ‘signal’ and ‘noise’. Thus, an individual with an imprecise visual representation of anger, which overlaps with the representation of sadness should find it difficult to discriminate between these two emotions. Our recent work tested this hypothesis by asking (non-autistic adult) participants to manipulate a dial to change the speed of a dynamic point light face (PLF) stimulus (depicting an actor speaking in a happy, angry or sad fashion) until it moved at the speed they typically associated with an angry, happy or sad expression. Thus, providing us with an estimate of the speed of participants’ internal visual representations of emotional expressions. Participants also completed an emotion recognition task in which they rated the extent to which PLF stimuli depicted different emotional expressions. Although we did not confirm a role for differentiation in emotion recognition, we did find (across two samples with a total N = 281) that adults with less precise emotion representations typically exhibited lower emotion recognition accuracy scores^17,19^. Thus, signal detection theory highlights two features of visual emotion representations that may be important in emotion recognition: (1) the precision, (2) the differentiation of these visual representations. Our empirical work to date has confirmed an important role for precision.

In addition to precision, our previous work showed that the general ability to match two images also plays an important role in emotion recognition. We theorized that to have superior emotion recognition, one may need to have (a) precise representations of facial expressions, *and* (b) the ability to *match* incoming expression stimuli to internal representations. To test matching, we asked participants to alter the speed of a PLF until it matched the speed of a second PLF^17^. Across both a discovery and replication sample, we found an interaction between representational precision and matching ability. That is, for participants with a good ability to visually match two expressions, representational precision was less important for emotion recognition. In contrast, if participants had a poorer ability to match expressions, representational precision played an important role.

In a parallel literature, there is preliminary evidence that autistic individuals struggle to differentiate their own emotions^20^. Since autistic individuals may struggle to differentiate emotional *states* they may also struggle to differentiate *visual representations* of emotion. That is, autistic individuals may picture emotional expressions in their mind’s eye as more similar and overlapping than their non-autistic peers (e.g., the angry and sad expressions they imagine look very similar and are easily confused for one another). This is particularly plausible given that (non-autistic) individuals with less differentiated *experiences* of emotion typically have less differentiated *visual representations* too^17^. As mentioned previously, since overlapping ‘signal’ and ‘noise’ distributions may make it difficult to discriminate the ‘signal’ from the ‘noise’^18^, it may be that difficulties differentiating *visual representations* are responsible for emotion recognition differences in autism. However, research has not yet tested this idea.

In sum, recent work has begun to elucidate a number of factors that could account for individual differences in emotion recognition, including the precision and differentiation of visual emotion representations and visual matching ability. It follows that emotion recognition difficulties in autism could be due to differences in one, or many, of these factors. For instance, autistic individuals may have more imprecise and/or overlapping visual representations of emotional expressions. Unpacking this may help to explain why not all studies find differences between autistic and non-autistic people with respect to emotion recognition: perhaps some emotion recognition tasks rely more on either the precision or differentiation of visual emotions representations, or more on these representations in general, than others. For example, affect matching paradigms, in which participants judge whether two expressions show the same or different emotions may place less emphasis on visual emotion representations (as participants compare expressions that are *presented* to them sequentially or simultaneously) than labelling paradigms, where participants may have to compare to their visual representations in order to produce the correct emotion label.

The current study therefore, first, investigated whether autistic and non-autistic adults differed with respect to the precision and/or differentiation of their visual representations of emotion and their general matching abilities (in the speed domain), and second explored whether differences therein were related to individual differences in accurately recognizing emotional expressions. In our study, we also controlled for alexithymia – a subclinical condition wherein individuals experience difficulties in identifying their own emotions^21^ – to ensure that any differences between the groups relate to autism, and not to alexithymia, as has been found in previous work^22–25^.

Recent Bayesian accounts of autism propose another possible source of differences in emotion recognition in autism. According to Bayesian accounts, prior expectations bias the perception of incoming sensory information. With respect to emotion recognition, if one expects to observe a happy expression, one will attend more to features that generally signal happiness and less to features that tend to signal other emotions^26^. Bayesian theories of autism argue that autistic people are less affected by prior expectations than neurotypical people^27,28^ and place greater emphasis on incoming sensory information (see Ref.^29^). Therefore, for non-autistic people, expectations can bias the perception of expressions (i.e., incoming sensory stimuli) such that they better match visual representations of expected emotions. For autistic people the perception of expressions may be less affected by prior expectations, and therefore their perception of the incoming expression may be less biased towards their visual emotion representation. If it is the case that autistic individuals are less affected by their visual representations of emotion (relative to non-autistic people), we would expect emotion recognition accuracy to be predicted by the precision and differentiation of these representations to a lesser extent than for non-autistic individuals. Consequently, in addition to investigating whether autistic and non-autistic adults differ in terms of matching abilities, and the precision and/or differentiation of visual emotion representations, we also assessed the extent to which a number of different abilities were implicated in autistic and non-autistic emotion recognition.

## Results

To determine whether there are differences between autistic and non-autistic individuals in these abilities, the current study employed three tasks involving dynamic point light displays of angry, happy and sad facial expressions. The first task was an adapted version of our “ExpressionMap” task^17,30,31^ which uses a method of adjustment design. On each trial, participants were required to manipulate a dial to speed-up or slow-down PLF stimuli until they matched their visual representation of anger, happiness, or sadness. This task assesses how precise (by assessing variability, across trials, in attributed speed) and overlapping (via assessing the mean distance between emotions in terms of speed) participants’ visual emotion representations are. In the second task, known as the “Visual Matching task”^17^, participants were required to match the speed of a PLF to another displayed PLF. Since participants are provided with a visual representation to match to, they do not need to imagine anything, and therefore this task indexes visual matching ability independent of imagination ability. Finally, we used our previously validated task^8,32^ to index emotion recognition ability. On each trial, participants viewed an angry, happy, or sad PLF and rated the extent to which the expression looked angry, happy and sad on visual analogue scales. Emotion recognition accuracy was calculated as the correct emotion rating minus the mean of the two incorrect emotion ratings.

In the following section, we (1) compare autistic and non-autistic participants on the precision and differentiation of visual emotion representations, matching abilities, and emotion recognition, and (2) determine whether the same processes are implicated in autistic and non-autistic emotion recognition.

### Analyses comparing autistic and non-autistic participants

First, to compare the precision of visual emotion representations (as measured by the ExpressionMap task) across participant groups, we conducted a linear mixed effects model with representational precision as the dependent variable, emotion (angry, happy, sad), group (autistic vs non-autistic), the interaction between emotion and group [independent variables], age, sex, non-verbal reasoning ability and alexithymia [control variables] as predictors, and subject number as a random intercept. This revealed that there was a significant main effect of emotion [F(2,176) = 87.13, *p *< 0.001]: precision scores were highest for sad [mean(standard error of the mean; SEM) = − 0.52(0.03)], followed by happy [mean(SEM) = − 0.68(0.04)], followed by angry expressions [mean(SEM) = − 0.91(0.04)]. In addition, both age [F(1,83) = − 18.23, *p *< 0.001], and non-verbal reasoning [F(1,83) = 18.10, *p *< 0.001] predicted representational precision. Most importantly, however, we identified a main effect of group [F(1,83) = 6.25, *p *= 0.014]: in contrast to our hypothesis, the autistic participants [mean(SEM) = − 0.64(0.04)] exhibited significantly higher precision than the non-autistic [mean(SEM) = − 0.77(0.04)] participants, suggesting that autistic individuals have more precise visual representations of emotion. The emotion x group interaction [*p *= 0.594], sex [*p *= 0.207], and alexithymia [*p *= 0.469] were not significant predictors of representational precision.

Next, to compare the distances between emotion representations across participant groups, we constructed a linear mixed effects model with distance as the dependent variable, emotion pair (angry-happy, angry-sad, happy-sad), group (autistic, non-autistic), the interaction between emotion pair and group [independent variables], age, sex, non-verbal reasoning, and alexithymia [control variables] as predictors, and subject number as a random intercept. In line with the results from our previous study^30^, this analysis found that there was a significant main effect of emotion [F(2,176) = 74.31 *p *< 0.001]: the distance between angry and sad emotion representations was largest [mean(SEM) = 2.25(0.11)], followed by the distances between angry and happy [mean(SEM) = 1.21(0.09)] and happy and sad [mean(SEM) = 1.14(0.07)] representations. There was no main effect of group [*p *= 0.117], nor an interaction between emotion pair and group [*p *= 0.317], suggesting that autistic and non-autistic individuals do not significantly differ in the differentiation of visual emotion representations. Finally, age [*p *= 0.080], sex [*p *= 0.174], non-verbal reasoning [*p *= 0.390] and alexithymia [*p *= 0.594] did not predict the distance between emotion representations.

Next, to compare the matching difficulty of the autistic and non-autistic participants, we ran a linear mixed effects model of matching difficulty as a function of emotion (angry, happy, sad), group (autistic, non-autistic), the emotion x group interaction [independent variables], age, sex, non-verbal reasoning, and alexithymia [control variables] with subject number as a random intercept. This analysis revealed that non-verbal reasoning ability was a significant negative predictor of matching difficulty [F(1,83) = − 15.75, *p *< 0.001]: those with higher non-verbal reasoning had a greater ability to match two visually displayed expressions on speed. Importantly, there was no significant main effect of group [*p *= 0.255] or an emotion x group interaction [*p *= 0.795], indicating that the autistic and non-autistic individuals had similar matching ability across all emotions. There was also no significant main effect of emotion [*p *= 0.058]. Age [*p *= 0.188], sex [*p *= 0.388], and alexithymia [*p *= 0.149] were also not significant predictors of matching difficulty.

Finally, we constructed a linear mixed effects model of emotion recognition accuracy (as measured by the PLF emotion recognition task) as a function of emotion (angry, happy, sad), spatial level (50%, 100%, 150% spatial exaggeration), kinematic level (50%, 100%, 150% speed), group (autistic, non-autistic), the interaction between these variables [independent variables], age, sex, non-verbal reasoning, and alexithymia [control variables], with subject number as a random intercept. This revealed that there was no significant main effect of group or any significant interactions with group (all *p *> 0.05). Therefore, the autistic and non-autistic participants exhibited comparable levels of accuracy across different emotions, speeds, and levels of spatial exaggeration. The remaining results from this analysis are reported in Supplementary Information A as they are outside the scope of the current study.

### Determining the contributors to autistic and non-autistic emotion recognition

To determine the relative importance of our variables of interest for autistic and non-autistic emotion recognition, we conducted a random forests analysis^33^ in each group using the *Boruta*^34^ wrapper algorithm (version 7.7.0; as in^17^). Random forest regression is a supervised machine learning technique that constructs a large number of decision ‘trees’, each predicting a continuous outcome variable with a collection of factors, and then aggregates these predictions into one final result (by taking a mean of the predictions from the individual tress). The Boruta wrapper algorithm starts by randomly shuffling each predictor variable and adding these shuffled variables (termed “shadow features”) to the dataset. Following this, across many iterations (here, 1500), the algorithm trains a random forest regression model on all the predictor variables, as well as their shuffled copies (i.e., “shadow features”), and classifies a variable as *important* (i.e., useful for predicting a target variable) when its importance score is higher than the highest importance of a shadow feature (termed “shadowMax” in the analysis; see Ref.^35^ for an accessible summary of the Boruta wrapper algorithm). In this analysis, our outcome variable was mean accuracy and our predictor variables were total AQ score, total TAS score, the AQ and TAS subscales (i.e., AQ Social Skills, AQ Attention Switching, AQ Attention to Detail, AQ Communication, AQ Imagination, TAS Difficulties Describing Feelings, TAS Difficulties Identifying Feelings, and TAS Externally Oriented Thinking), non-verbal reasoning ability, age, mean representational precision, mean distance, and the interaction between representational precision and matching (‘representation matching’)(thus following similar procedures to^17^).

**Figure 1:**
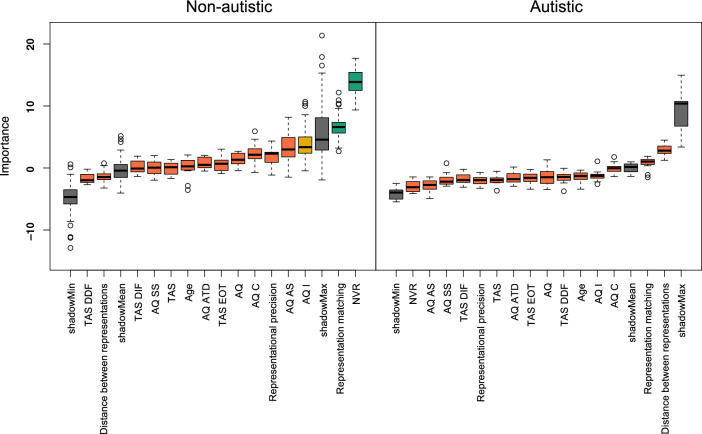
Random forest variable importances for non-autistic (left) and autistic (right) participants. Variable importance of all 15 features entered into the Boruta random forest, displayed as boxplots. Box edges denote the interquartile range (IQR) between the first and third quartile; whiskers denote 1.5 * IQR distance from box edges; circles represent outliers outside of 1.5 * IQR above and below box edges. Box color denotes decision: Green – confirmed, yellow = tentative, red = rejected; grey = meta-attributes shadowMin, shadowMax and shadowMean (minimum, maximum and mean variable importance attained by shadow features).

For the non-autistic participants, of the 15 variables tested, two were confirmed *important*, one was *tentative*, and 12 were confirmed *unimportant.* Figure 1 (left) illustrates that the representational precision x matching interaction and non-verbal reasoning ability were classed as important for non-autistic emotion recognition, with mean importance scores of 6.57 and 13.88 respectively. AQ Imagination score was classified as tentatively important with a mean importance score of 3.78. All other variables were deemed unimportant. In contrast, for the autistic participants all 15 of the tested variables were confirmed *unimportant* (see Fig. 1 right).

Following this, to verify the results from our random forests analysis for non-autistic individuals, we constructed a Bayesian linear regression model of accuracy as a function of non-verbal reasoning, the interaction between representational precision and matching (‘representation matching’), and AQ Imagination score. The strongest model that emerged from this analysis included just non-verbal reasoning ability and the representational precision x matching interaction as predictors of emotion recognition accuracy (and not AQ I score) [BF_10_ = 149.64, R^2^ = 33.5%]. According to the model, there was very strong evidence that both of these factors contribute to emotion recognition accuracy for non-autistic individuals. When this analysis was conducted with autistic participants, there was moderate evidence that these variables did not predict emotion recognition accuracy [i.e., the null hypothesis; BF_10_ = 0.15, R^2^ = 1.00%], thus confirming the results from our previous analysis.

## Discussion

The current study compared autistic and non-autistic adults on features of visual representations thought to be implicated in emotion recognition (e.g., precision and differentiation of visual emotion representations, general matching ability), and investigated the contribution of these factors to emotion recognition in both groups. We found that the autistic participants had more precise visual emotion representations (in the speed domain) across all three emotions, thus contradicting our expectations. In addition, we identified that there were no significant differences between groups in emotion recognition accuracy. This was true across all levels of the spatial and kinematic manipulations. This finding contradicts previous studies identifying group differences in emotion recognition (e.g.,^8,10,36,37^) and instead supports literature suggesting emotion recognition performance is comparable between autistic and non-autistic people (e.g.,^22,38–41^). Furthermore, there were no significant differences between groups in differentiation – as indexed by the distance between emotion representations—or matching ability. Hence, although autistic individuals may have less distinct emotional experiences (as in^20^), they have comparably distinct visual representations (at least in the speed domain) to their non-autistic counterparts. In sum, these results show that the autistic participants had more precise visual emotion representations (in terms of speed), but that this did not confer any benefit in terms of accuracy on our task which indexed emotion recognition from dynamic stimuli (although it is possible that having more precise visual emotion representations benefits autistic individuals on other types of tasks).

To determine the relative importance of various abilities (e.g., precision of representations, distance between representations, general matching ability) and clinically relevant individual differences (e.g., non-verbal reasoning, AQ, TAS) to autistic and non-autistic emotion recognition, we conducted random forest analyses employing the Boruta wrapper algorithm. Whilst for non-autistic individuals, non-verbal reasoning ability and the interaction between representational precision and matching were classified as *important*, and AQ Imagination score was classified as *tentatively important* for emotion recognition, no variables were deemed important for autistic emotion recognition. Of particular note, none of the variables corresponding to features of emotion representations contributed to autistic emotion recognition (i.e., precision of representations, distance between representations, the representational precision x matching interaction). That is, these factors were no better than randomly shuffled data at predicting emotion recognition accuracy. Thus, aside from precision there were minimal differences between the groups (matching, distinctness and accuracy did not significantly differ); nevertheless, there were differences in the way these variables were related such that the autistic participants did not exhibit the predictive relationship between features of representations and emotion recognition accuracy that is exhibited by non-autistic people. These results suggest differences in the psychological mechanisms underpinning emotion recognition from dynamic stimuli in autism.

One possible mechanistic difference is that autistic individuals may not be ‘using’ their (precise) emotion representation (or ‘using’ them to a lesser extent) to help them recognize emotional expressions. This idea aligns well with Bayesian accounts of autism which posit that autistic individuals are less influenced by priors than non-autistic people (see Ref.^29^). To date, there is mixed evidence in relation to these Bayesian accounts, with some studies suggesting weaker prior influences, others suggesting no differences, and a handful suggesting larger prior influences in autism (see Ref.^42^). Furthermore, there is variance across domains: for ‘social priors’, the evidence is almost evenly split between suggesting weaker prior influences and no differences, while for simpler perceptual priors there are usually no differences between autistic and non-autistic people (see Ref.^42^). One issue that is unresolved in this field is the question of whether autistic individuals possess *weaker* priors and/or whether autistic individuals are *less influenced* by priors. The two are orthogonal to each other so that, in theory one could have strong priors but nevertheless be weakly influenced by them. Our results raise the hypothesis that—at least in the domain of emotion recognition – autistic individuals have strong priors (i.e., precise emotion representations in the speed domain) but are, nevertheless weakly influenced by them (i.e., the relationship between the priors and emotion recognition accuracy is minimal). Future research is required to test this hypothesis.

If it is true that autistic individuals are less guided by their visual emotion representations, we might expect these individuals to perform better on tasks that do not require a comparison between incoming facial expressions and internal templates. For example, they may perform better on affect matching paradigms, wherein participants have to judge whether two expressions show the same or different emotions (i.e., differentiate emotional expressions that are presented to participants sequentially or simultaneously), rather than labelling paradigms, where participants may have to compare to their visual representations in order to produce the correct emotion label. In line with this, whilst numerous studies employing matching paradigms show comparable emotion recognition performance between autistic and non-autistic people (e.g.,^38,43–45^), those employing labelling paradigms often document differences between these groups (e.g.,^46–53^). As such, our findings may help disentangle mixed findings regarding emotion recognition in autism by suggesting that autistic individuals may have particular difficulties on tasks that mandate comparison to their internal templates.

If autistic individuals are less guided by their visual representations of emotion, how are they able to achieve high levels of accuracy on our emotion recognition task? One plausible explanation is that autistic individuals have developed compensatory strategies that allow them to achieve comparable accuracy to non-autistic participants on certain tasks (e.g., in the current study; see Ref.^54^). The nature of these compensatory strategies may vary from person to person, but one possibility is that autistic people use explicit cognitive or verbally mediated strategies to help them recognize emotions (in contrast to more automatic processing in non-autistic individuals^54,55^). Here, rather than automatically comparing their visual emotion representations to incoming facial expressions, the autistic participants may instead follow a “rule-based strategy” where they assess the degree to which the expression matches a list of features they have learnt to be associated with anger (e.g., “furrowed eye-brow”, “fast-moving”, etc.), happiness (“lip raising”, “teeth showing”, etc.), and sadness (“downturned mouth”, “slow-moving”, etc.), along with other emotions^55^.

If autistic participants are using an alternative, cognitive or verbal, strategy we might expect emotion recognition performance to be more related to general cognitive or verbal ability for autistic people than for non-autistic people. Supporting this idea, studies have found that mental age^56^, and receptive and expressive language^57^ predict emotion recognition ability in autistic, but not non-autistic, children. Concurrently, if autistic individuals are less reliant on visuo-spatial cues (such as visual emotion representations), we might also expect non-verbal reasoning ability to be less associated with emotion recognition performance in the autistic than non-autistic group. In line with this, here we found that non-verbal reasoning ability was a significantly stronger predictor of emotion recognition accuracy [z = − 2.25, *p *< 0.05] for the non-autistic [t = 3.88, *p *< 0.001, BF_10_ = 74.16, R^2^ = 25.9%], than autistic [t = − 0.46, *p *= 0.650, BF_10_ = 0.321, R^2^ = 0.5%], participants. Third, if it is true that autistic individuals employ more effortful cognitive/verbally mediated mechanisms to recognize emotions (rather than a more automatic processing style), this could explain why autistic individuals typically exhibit longer emotion recognition response latencies than non-autistic individuals (e.g.,^46,58–65^; though note there could be other explanations for longer response latencies). Here, the PLF stimuli were presented for relatively long durations (approximately 6 s on average), thus potentially providing the autistic participants sufficient time to employ their compensatory strategies (and hence they were able to reach comparable accuracy scores). Further research is necessary to confirm whether autistic individuals adopt a rule-based strategy to read emotional facial expressions.

### Limitations

The results of the current study are informative with respect to understanding the emotion representations and emotion recognition of autistic and non-autistic individuals from facial motion cues alone. However, since many features of expressions are involved in emotion processing, such as shading/depth^66^ and pigmentation/colouring^67^, one should be cautious to assume that our findings generalize to full dynamic emotional expressions (e.g., full video recordings of facial expressions). It could be, for instance, that the precision of emotion representations and matching ability are important for autistic emotion recognition for full dynamic expressions, but not point-light displays. However, since our study was motivated by the observation of group differences in emotion recognition^8^, and links discovered between emotion representations and emotion recognition from facial motion cues alone (as in Ref.^17,19^), it was crucial to our overall research question that we used PLF stimuli in the current study. Although this was an active design choice, motivated by previous research demonstrating a causal role of speed cues in emotion recognition^32^ and other a priori hypotheses (see Ref.^30^), in future work we will develop our paradigms to encompass other spatiotemporal emotion cues. Thus, facilitating comparisons of visual emotion representations between autistic and non-autistic individuals with respect to other cues such as the degree of spatial exaggeration, movement onset/offset, texture and colour.

It is also important to address the limitations of our study with respect to generalizability. Notably, the participants in our sample were predominantly white (86.67%; see Supplementary Information B), highly educated (see Supplementary Information C), English-speaking individuals from highly developed countries. As such, our sample may not be representative of those with lower levels of education or intellectual disabilities, or those from different racial, ethnic, cultural, or socioeconomic backgrounds. With respect to the former, whilst autistic individuals with average to high IQs often have comparable emotion recognition performance (e.g.,^22,39–41^), those with co-occurring intellectual disabilities appear to struggle with emotion recognition (e.g.,^56,68,69^), relative to IQ or mental age-matched comparison groups (though see Ref.^70^). Hence, we may not have found emotion recognition difficulties here due to our autistic participants possessing high levels of intelligence (as demonstrated by their high level of education). With respect to the latter, since the participants in our sample are predominantly from developed countries, where emotion recognition interventions are increasingly being offered to autistic individuals (e.g.,^37,71,72^), it may be that some of our autistic participants have undergone training in the past, thus improving their emotion recognition scores. Hence, our findings may not represent the emotion recognition performance of autistic individuals from less developed countries. Future studies should aim to dismantle barriers to inclusion to boost the representativeness of their samples, thus allowing us to identify whether specific subgroups of autistic individuals (e.g., those with intellectual disabilities) have difficulties with emotion recognition (and other emotion processes).

## Conclusion

The current study aimed to compare autistic and non-autistic participants on features of their emotion representations, and determine whether the same processes are implicated in autistic and non-autistic emotion recognition. Using a method of adjustment design, we found that autistic individuals had more precise visual emotion representations than their non-autistic counterparts (in the speed domain). That is, the autistic participants were more precise (i.e., consistent) in the speeds they attributed to angry, happy and sad facial expressions across repetitions. Nevertheless, this enhanced precision did not confer any benefit for their emotion recognition. Whilst for non-autistic people, non-verbal reasoning and the interaction between precision of emotion representations and matching ability predicted emotion recognition, no variables contributed to autistic emotion recognition. These findings highlight the possibility that autistic individuals are less guided by their emotion representations (a form of prior). Future research is necessary to identify what traits, processes, and strategies are implicated in autistic emotion recognition.

## Method

This study was approved by the Science, Technology, Engineering and Mathematics (STEM) ethics committee at the University of Birmingham (ERN_16-0281AP9D) and was conducted in accordance with the principles of the revised Helsinki Declaration. Informed consent was obtained from all participants.

### Participants

A total of 45 autistic and 45 non-autistic participants were recruited from the Birmingham Psychology Autism Research Team (B-PART) database, the Centre for Autism Research Oxford database, and Prolific. All participants in the autistic group had previously received a clinical diagnosis of ASD from an independent clinician. As expected, the autistic participants had significantly higher AQ scores than the non-autistic participants (see Table 1.).

**Table 1:** Means, standard deviations, and group differences of participant characteristics.

Variable	Autistic (n = 45)	Non-autistic (n = 45)	Significance
Sex	30 Female, 14 Male, 1 Prefer not to say	26 Female, 19 Male	*p *= 0.360
Age	35.51 (14.06)	34.87 (9.01)	*p *= 0.398
NVR	65.83%(15.31%)	63.70% (15.20%)	*p *= 0.255
AQ-50	37.31(7.64)	21.44 (7.34)	*p *< 0.001
TAS-20	64.60(12.46)	57.49 (11.99)	*p *= 0.004

In the central columns, means are followed by standard deviation in parentheses. Note that age is in years.

The sample size was based on an a priori power analysis conducted using G*Power^73^, which focuses on replicating the group-difference^8^ in recognition accuracy (between autistic and non-autistic individuals) for angry videos at the normal spatial and speed level. Using data from Keating et al.,^8^, 25 participants are required in each group in order to have 80% power to detect an effect size of 0.719 (Cohen's d) at alpha level 0.05 for this group-difference in accuracy. Since Button and colleagues^74^ argue that sample size calculations are likely to be highly optimistic, we recruited 45 participants in each group in order to ensure we obtained adequate power.

### Procedures

Following participatory research guidelines^75,76^, prior to conducting both studies, a group of individuals from the autism community (from the Birmingham Psychology Autism Research Team Consultancy Committee) provided feedback on our research (e.g., about task design and instructions, frequency of breaks, and suggested routes for dissemination, etc.). Following this consultation, we made a number of changes (e.g., added instruction videos for the ExpressionMap and Visual Matching task to promote understanding and accessibility) before starting to recruit participants.

Participants completed demographics questions, followed by the 50-item Autism Quotient^77^, and the 20-item Toronto Alexithymia Scale^78^. Following this, participants completed three tasks that employed dynamic point light displays (a series of dots that convey biological motion) of angry, happy and sad facial expressions (PLFs). Participants completed the ExpressionMap paradigm, followed by the Visual Matching task, followed by the PLF Emotion Recognition task. Finally, participants completed the Matrix Reasoning Item Bank (MaRs-IB;^79^). Within each task, participants were encouraged to take regular breaks in between blocks. All parts of the study were completed online in one sitting. Together, these questionnaires and tasks took approximately two hours and 30 min to complete.

### Materials and stimuli

#### The Autism Quotient

The level of autistic traits of participants was assessed via the self-report Autism Quotient^77^. This 50-item questionnaire is scored on a range from 0 to 50, with higher scores representing higher levels of autistic characteristics. The AQ assesses five different domains relevant for autistic traits: attention switching, attention to detail, communication, social skill and imagination. The AQ has been widely used to assess autistic traits in both the autistic and non-autistic people (e.g.,^80–82^), and has high internal consistency (α ≥ 0.70^83^, current study α = 0.92) and test–retest reliability (r ≥ 0.8;^83^).

#### The Toronto Alexithymia Scale

The level of alexithymic traits of participants was measured via the 20-item Toronto Alexithymia Scale^78^. This self-report tool comprises 20 items rated on a five-point Likert scale, ranging from 1, strongly disagree, to 5, strongly agree. Total scores on the scale range from 20 to 100, with higher scores indicating higher levels of alexithymic traits. The TAS-20 is the most popular tool for assessing alexithymia and has high internal consistency (α ≥ 0.70^78,84^, current study α = 0.84) and test–retest reliability (r ≥ 0.7;^78,84^).

#### ExpressionMap

**Figure 2:**
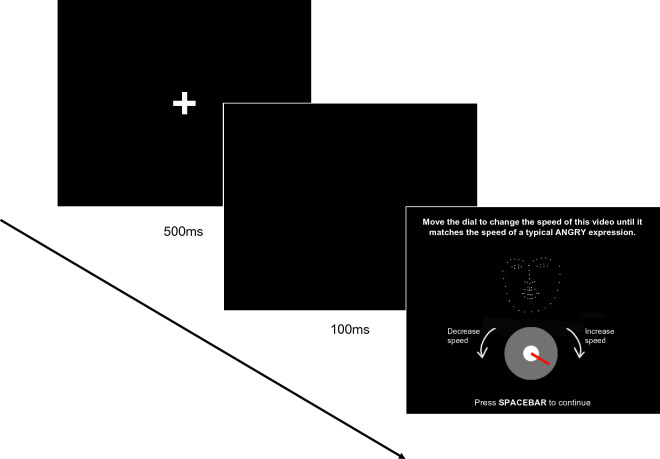
Example of one trial in the ExpressionMap paradigm. The fixation cross was presented for 500 ms at the start of each trial, followed by a blank screen for 100 ms. The instructions, PLF expression and dial remained on screen until participants pressed the spacebar to continue.

Before completing the ExpressionMap task, participants watched an instruction video guiding them through one trial. During the task, on each trial, participants were presented with a PLF stimulus video (on average, approximately 6 s in length) which looped such that it played continuously. On each trial, this stimulus video started at a random speed. Participants were instructed to “move the dial to change the speed of this video until it matches the speed of a typical ANGRY/HAPPY/SAD expression” (note that participants were only asked to change the speed of the expression to match the emotion that was displayed in the stimulus video, i.e., on a trial where an angry facial expression was presented, participants were only asked to manipulate the speed of the video so that it matched the speed of a typical angry expression; see Fig. 2 for trial structure). Consequently, participants were matching the speed of the displayed PLF to their visual representation of that expression. Participants could change the speed of the video by moving a dial clockwise to increase the speed of the animation or anticlockwise to decrease the speed of the animation. The minimum and maximum point on the dial corresponded with 25% and 300% of the recorded speed respectively (though note that participants were not informed about this). Once participants were satisfied that the speed of the video matched that of a typical angry/happy/sad expression, they could press the spacebar to continue. There was no time limit for participants to respond on each trial. Participants were shown four repetitions of each PLF stimulus video across four actors. This resulted in 16 videos per emotion (4 actors × 4 repetitions × 3 emotions = 48 trial in total). Participants completed three practice trials (one for each emotion at 100% starting speed) and then the 48 randomly ordered experimental trials across three blocks. Participants were encouraged to take breaks between blocks.

The ExpressionMap task provides an index of the percentage speed attributed to each of the stimulus videos by participants (e.g., 130% speed). Following the procedures outlined in Keating, Sowden & Cook^30^, we calculated the true speed attributed to each of the PLFs (in pixels per frame) by multiplying the percentage speed attributed, divided by 100, with the speed of the actor’s facial movement in the original video. For example, for a trial in which a participant attributed 200% speed to a face moving at 2.5 pixels/frame, the true speed attributed to the expression would be 5 pixels/frame [i.e., $\left(200\div 100\right) \times 2.5$] (see Ref.^30^ for more information).

The ExpressionMap task operates on the premise that those with less precise representations of emotion will attribute more variable speeds to the expressions (than those with more precise representations; see Ref.^31^ for an accessible summary). Therefore, to index the precision of visual emotion representations, we took the standard deviation of the speeds attributed to one emotion for one actor (i.e., actor 1, angry expression) across the 4 video repetitions. Following this, we multiplied these variability scores by -1 so that our variable would represent precision. We then calculated mean representational precision for each of the emotions (angry, happy and sad) by taking a mean of the precision scores for each actor within an emotion (e.g., taking a mean of the precision scores for angry expressions across actors 1, 2, 3 and 4; see Supplementary Information D for justification of calculating representational precision in this way). Overall mean representational precision was calculated by taking a mean of the precision scores for the angry, happy and sad PLFs.

Finally, the ExpressionMap task also provides an index of the ‘distance’ between emotions, thus informing us how well differentiated participants’ visual representations of angry, happy and sad facial expressions are in the speed domain. To calculate distance scores, we subtracted the speed attributed to one emotion from the speed attributed to another and then took the absolute value of this number. For example, to calculate distance between happy and angry, we subtracted the speed attributed to happy from the speed attributed angry, and then took the absolute value. Mean distance was calculating by taking a mean of the scores for the angry-happy, angry-sad, and happy-sad distances.

#### Visual Matching task

**Figure 3:**
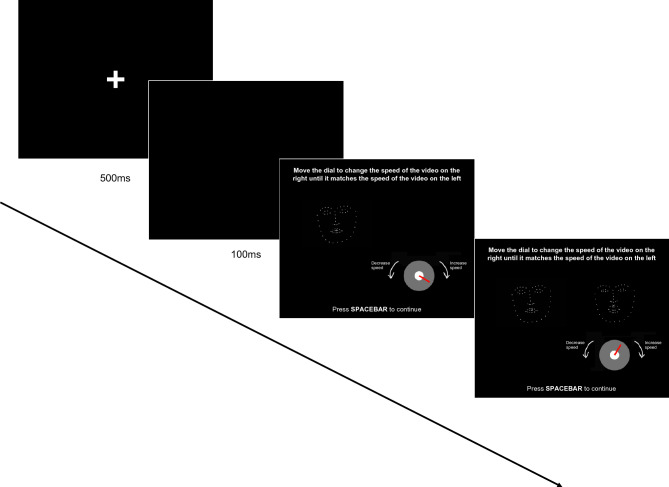
Example of one trial in the Visual Matching task. The fixation cross was presented for 500 ms at the start of each trial, followed by a blank screen for 100 ms. Next the instructions, the dial, and the PLF expression on the left-hand side appeared on screen. After the PLF expression on the left-hand side had played once, the PLF expression on the right-hand side appeared on screen. These expressions, along with the instructions and dial, remained on screen until participants pressed the spacebar to continue.

The Visual Matching task assesses how well participants can visually match the speed of one expression to another displayed expression. Before completing the task, participants watched an instruction video guiding them through one trial. Each trial began with a PLF stimulus video on the left-hand side of the screen. After this video had played once, the same PLF stimulus video also appeared on the right-hand side of screen, playing repeatedly at a fixed random speed. Participants were instructed to “move the dial to change the speed of the video on the right until it matches the speed of the video on the left” (see Fig. 3 for trial structure). Consequently, participants were visually matching the speed of one PLF to another. Participants could turn the dial clockwise to increase, and anticlockwise to decrease, the speed of the animation on the right. The minimum and maximum points on the dial corresponded with 25% speed and 300% of the recorded speed respectively (participants were not informed about this). Once the participant was satisfied that the speed of the animation on the right matched the speed of the animation on the left, they pressed spacebar to continue. Participants were shown four repetitions of each PLF stimulus video for each of the four actors. Each repetition had a different starting speed. In each full set of 16 (4 actors × 4 repetitions) stimulus videos for an emotion, the starting speed ranged from 50% to 200% of the recorded speed, in 10% increments. We selected these starting speeds to ensure that participants were able to visually match the two displayed expressions across a variety of speeds. Participants completed three practice trials (one for each emotion at 100% starting speed) and then the 48 randomly ordered experimental trials across three blocks. Participants were given the opportunity to take breaks between blocks.

The Visual Matching task provides an index of how well participants can visually match the speed of one expression to another. To calculate deviation scores, we subtracted the percentage speed attributed to the expression on the right from the percentage speed of the video on the left, and then took the absolute value of this deviation score as a measure of how far away the speeds of the two animations were (irrespective of whether they attributed too high or too low speed). Finally, we calculated mean deviation scores by taking a mean of all of the absolute deviation scores.

#### PLF Emotion Recognition task

**Figure 4:**
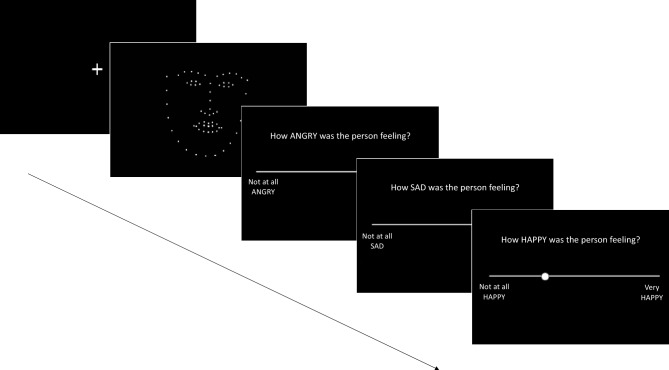
Example of one trial in the PLF Emotion Recognition Task (as in Keating et al.,^8^). The fixation cross was presented for 500 ms at the start of each trial. The average length of a stimulus video was approximately 7 s. Rating scales remained on screen until participants had rated the stimulus and pressed the space bar.

The PLF Emotion Recognition task was identical to that reported in Sowden et al.,^32^ and Keating et al.,^8^. Participants viewed dynamic PLFs, created from videos of four actors verbalizing sentences whilst posing three target emotions (angry, happy and sad). These PLFs have been adapted (see Ref.^32^ for further detail) to achieve three spatial movement levels, ranging from decreased to increased spatial movement (50%, 100% and 150% spatial movement), and three kinematic levels, ranging from reduced to increased speed (50%, 100% and 150% original stimulus speed). Each trial in this task began with the presentation of a (silent) PLF video displaying one of the 3 emotions, at one of the 3 spatial and 3 kinematic levels. After watching the video, participants were required to rate how angry, happy and sad the person was feeling on three visual analogue scales (presented in a random order) ranging from ‘Not at all angry/happy/sad’ to ‘Very angry/happy/sad’ (see Fig. 4 for trial structure). Each trial lasted approximately 25 s. Participants completed three practice trials and then 108 randomly ordered experimental trials (12 per condition) across three blocks. Participants were encouraged to take a break between blocks.

The three emotion rating responses for each trial were transformed into magnitude scores from 0 to 10 (with 0 representing a response of ‘Not at all’ and 10 representing ‘Very’) to three decimal places. Emotion recognition accuracy scores were calculated by subtracting the mean of the two incorrect emotion ratings from the correct emotion rating. For example, for a trial in which a happy PLF was displayed, the mean rating of the two incorrect emotions (angry and sad) was subtracted from the rating for the correct emotion (happy). Mean emotion recognition accuracy was calculated by taking a mean of accuracy scores across all emotions and levels of the spatial and kinematic manipulations.

#### The Matrix Reasoning Item Bank (MaRs-IB)

In this experiment, non-verbal reasoning ability was assessed via the Matrix Reasoning Item bank (see MaRs-IB;^79^). Each item in the MaRs-IB consists of a 3 × 3 matrix. In this matrix, eight of the nine available cells are filled with abstract shapes, and one cell in the bottom right-hand corner is left empty. Participants are required to complete the matrix by selecting the missing shape from four possible options. To correctly identify the answer, participants have to deduce relationships between the shapes in the matrix (which vary in shape, colour, size and position). When participants select an answer, they proceed to the next trial. If participants do not provide a response within 30 s, they proceed to the next trial without a response. The MaRs-IB assessment lasts eight minutes regardless of how many trials are completed. The MaRs-IB has acceptable internal consistency (Kuder-Richardson 20 ≥ 0.7) and test–retest reliability (r ≥ 0.7;^79^).

### Statistical analyses

All frequentist analyses were conducted using R Studio (version 2021.09.2) and all Bayesian analyses were conducted using JASP (version 0.16). For all frequentist analyses, we used a significance threshold of *p *= 0.05 to determine whether to accept or reject the null hypothesis. The frequentist approach was supplemented with the calculation of Bayes Factors, which quantify the relative evidence for one theory or model over another. For all Bayesian analyses, we followed the classification scheme used in JASP^85^, in which BF_10_ values between one and three reflect weak evidence, between 3 and 10 as moderate evidence and greater than 10 as strong evidence for the *experimental* hypothesis. In addition, BF_10_ values between 1 and 1/3 reflect weak evidence, between 1/3 and 1/10 as moderate evidence, and smaller than 1/10 as strong evidence for the *null* hypothesis respectively^85^.

## Supplementary Information

Supplementary Information.

## Data Availability

The data files corresponding to this study are available online at https://doi.org/10.17605/OSF.IO/UQY3K. The tasks used in the current study are openly available online at https://app.gorilla.sc/openmaterials/447800.
