# Supplementary material for: Autistic adults exhibit highly precise representations of others’ emotions but a reduced influence of emotion representations on emotion recognition accuracy
Source: Sci Rep. 2023 Jul 22;13:11875. doi: 10.1038/s41598-023-39070-0 (PMC10363153; doi:10.1038/s41598-023-39070-0)
Supplement: Supplementary file 1 — Supplementary Information. [file 41598_2023_39070_MOESM1_ESM.docx]

**Supplementary Information for:**

**Autistic adults exhibit highly precise representations of others’ emotions but a reduced influence of emotion representations on emotion recognition accuracy**

Connor T. Keating^1^*, Eri Ichijo^2^, Jennifer L. Cook^1^

^1^School of Psychology, University of Birmingham

^2^Department of Experimental Psychology, University of Oxford

**Supplementary Information A- The full results from our linear mixed effects model of accuracy**

As stated in the main manuscript, we constructed a linear mixed effects model of emotion recognition accuracy (as measured by the PLF emotion recognition task) as a function of emotion (angry, happy, sad), spatial level (50%, 100%, 150% spatial exaggeration), kinematic level (50%, 100%, 150% speed), group (autistic, non-autistic), the interaction between these variables (independent variables), age, sex, non-verbal reasoning, and alexithymia (control variables) as predictors, and subject number as a random intercept. This analysis revealed a significant main effects of emotion [F(2,2318) = 112.12, p < .001], spatial level [F(1,2318) = 162.37, p < .001], and kinematic level [F(1,2318) = 10.60, p = .001], which were qualified by emotion x spatial [F(2,2318) = 36.75, p < .001], and emotion x kinematic [F(2,2318) = 18.60, p < .001] interactions. Unpacking the emotion x spatial interaction demonstrated that whilst emotion recognition accuracy improved with *increasing* spatial exaggeration for anger and happiness, it improved with *decreasing* spatial exaggeration for sadness (as in Sowden et al., 2021). Similarly, unpacking the emotion x kinematic interaction revealed that whilst emotion recognition accuracy improved with *increasing* speed for anger and happiness, it improved with *decreasing* speed for sadness (as in Sowden et al., 2021). Finally, we also identified that non-verbal reasoning ability was a significant predictor of emotion recognition accuracy [F(1,83) = 5.84, = .018]: those higher in non-verbal reasoning ability had better emotion recognition.

**Supplementary Information B –** Participants’ ethnicities

| **Racial Group** | **Ethnic Group** | **N** | **%** |
| --- | --- | --- | --- |
| White | White English/ Welsh/ Scottish/ Norther Irish/ British | 45 | 50.0% |
|  | White European | 6 | 6.7% |
|  | White Irish | 3 | 3.3% |
|  | White Portuguese | 2 | 2.2% |
|  | White Greek | 2 | 2.2% |
|  | White Turkish | 2 | 2.2% |
|  | White Polish | 2 | 2.2% |
|  | White Caucasian | 2 | 2.2% |
|  | White Slavonic | 1 | 1.1% |
|  | White American | 1 | 1.1% |
|  | White Dutch | 1 | 1.1% |
|  | White Hungarian | 1 | 1.1% |
|  | White South African | 1 | 1.1% |
|  | White Ukrainian | 1 | 1.1% |
|  | White Australian | 1 | 1.1% |
|  | White New Zealand | 1 | 1.1% |
|  | White Honduran | 1 | 1.1% |
|  | White Scandinavian | 1 | 1.1% |
|  | Mixed/Multiple ethnic groups: White Lithuanian/Finish/Irish | 1 | 1.1% |
|  | Mixed/Multiple ethnic groups: White British and Irish | 1 | 1.1% |
|  | Mixed/Multiple ethnic groups: White regions | 1 | 1.1% |
|  | Mixed/Multiple ethnic groups: White Sardinian, Italian, Ashkenazi | 1 | 1.1% |
| Asian | Asian Indian | 2 | 2.2% |
|  | Chinese | 1 | 1.1% |
| Black | Black African | 2 | 2.2% |
|  | Black Afrikaans | 1 | 1.1% |
|  | Black British | 1 | 1.1% |
|  | Black South African | 1 | 1.1% |
| Latino/Latina/Latinx | Latino | 1 | 1.1% |
| Mixed/Multiple ethnic groups | Mixed/Multiple ethnic groups: White and Asian | 1 | 1.1% |
|  | Mixed/Multiple ethnic groups: White and Native American | 1 | 1.1% |
|  | Mixed/Multiple ethnic groups: White, Black Caribbean and Hispanic | 1 | 1.1% |

**Supplementary Information C –** Participants’ level of education.

| **Highest level of education** | **N** | **%** |
| --- | --- | --- |
| Secondary School | 10 | 11.1% |
| Sixth Form or College | 19 | 21.1% |
| Diploma or equivalent level | 8 | 8.9% |
| Undergraduate degree or equivalent level | 27 | 30.0% |
| Master’s degree or equivalent level | 24 | 26.7% |
| PhD or equivalent level | 2 | 2.2% |

**Supplementary Information D – Explanation for why we calculated representational precision for each actor independently and then averaged across.**

There is evidence that individuals have identity-dependent (i.e., actor-specific) visual representations of emotion (see [Fox & Barton, 2007](https://www.sciencedirect.com/science/article/abs/pii/S0006899306029210?via%3Dihub); [Skinner & Benton, 2012](https://jov.arvojournals.org/article.aspx?articleid=2121059)). This idea is logical: if you see someone with furrowed eyebrows, usually you would interpret them as angry, however, if you are aware that the actor *naturally* has angular eyebrows, you might not interpret them as such. Since individuals tend to build actor-specific expression representations, the most logical approach for calculating precision is based on one actor’s expressions at a time, and then averaging across (rather than taking a standard deviation across every repetition of all four actor’s expressions for each emotion).

If we were to take a standard deviation across all four actor’s angry expressions, our results would be confounded by the extent to which participants have actor-specific representations. To illustrate this point, imagine that participant A has very precise visual representations of anger that tend to be actor-specific (in terms of speed). This participant may be likely to attribute 1.1 units of speed, 1.3 speed, 1.5 speed, and 1.7 speed to actor 1, and 3.5, 3.6, 3.8, and 3.9 to actor 2. It is reasonable for this participant to attribute different speeds to each actor, as there may be differences in the spatial configuration and speed of facial features between actors, and these cues heavily influence emotion judgements (see [Diego-Mas et al., 2020](https://journals.sagepub.com/doi/full/10.1177/2041669520961123); [Sowden et al., 2021](https://pubmed.ncbi.nlm.nih.gov/33661668/)). To give an example, it may be that actor 1 naturally has a more furrowed brow, meaning that they appear angry at lower speeds than actor 2. Based on their speed attributions, we can see that participant A has highly precise visual representations for each actor, but the speed of the representations differs between actors. In comparison, consider participant B that has imprecise visual representations of facial expressions, that are not individualized across actors. This individual may attribute 1.5 speed, 1.9 speed, 2.3 speed and 3.8 speed to actor 1, and 1.3 speed, 2.1 speed, 3.0 speed and 4.1 speed to actor 2 (and thus is imprecise across both actors). If we were to take a standard deviation across actors (as suggested by the reviewer as a potential alternative method), participant A would score -1.25, indicating low precision, and participant B would score -1.04, indicating higher precision. Hence, participant B who has considerably less precise visual representations would score higher than participant A in precision. However, using our method for calculating precision, participant A scores -0.22, indicating high precision, and participant B scores -1.12, indicating low precision. Hence, our method is a more valid way of measuring the precision of visual emotion representations.
